# Human Aquaporin-5 Facilitates Hydrogen Peroxide Permeation Affecting Adaption to Oxidative Stress and Cancer Cell Migration

**DOI:** 10.3390/cancers11070932

**Published:** 2019-07-03

**Authors:** Claudia Rodrigues, Catarina Pimpão, Andreia F. Mósca, Ana S. Coxixo, Duarte Lopes, Inês Vieira da Silva, Per Amstrup Pedersen, Fernando Antunes, Graça Soveral

**Affiliations:** 1 Research Institute for Medicines (iMed.ULisboa), Faculty of Pharmacy, Universidade de Lisboa, 1649-003 Lisboa, Portugal; 2 Department of Biochemistry and Human Biology, Faculty of Pharmacy, Universidade de Lisboa, 1649-003 Lisboa, Portugal; 3 Department of Biology, University of Copenhagen, Universitetsparken 13, DK-2100 Copenhagen OE, Denmark; 4 Centro de Química e Bioquímica, Centro de Química Estrutural e Departamento de Química e Bioquímica, Faculdade de Ciências, Universidade de Lisboa, 1749-016 Lisboa, Portugal

**Keywords:** aquaporin, permeability, hydrogen peroxide, oxidative stress, cell migration, cancer

## Abstract

Reactive oxygen species (ROS), including H_2_O_2_, contribute to oxidative stress and may cause cancer initiation and progression. However, at low concentrations, H_2_O_2_ can regulate signaling pathways modulating cell growth, differentiation, and migration. A few mammalian aquaporins (AQPs) facilitate H_2_O_2_ diffusion across membranes and participate in tumorigenesis. AQP3 and AQP5 are strongly expressed in cancer tissues and AQP3-mediated H_2_O_2_ transport has been related to breast cancer cell migration, but studies with human AQP5 are lacking. Here, we report that, in addition to its established water permeation capacity, human AQP5 facilitates transmembrane H_2_O_2_ diffusion and modulates cell growth of AQP5-transformed yeast cells in response to oxidative stress. Mutagenesis studies revealed that residue His173 located in the selective filter is crucial for AQP5 permeability, and interactions with phosphorylated Ser183 may regulate permeation through pore blockage. Moreover, in human pancreatic cancer cells, the measured AQP5-mediated H_2_O_2_ influx rate indicates the presence of a highly efficient peroxiporin activity. Cell migration was similarly suppressed by AQP3 or AQP5 gene silencing and could be recovered by external oxidative stimuli. Altogether, these results unveiled a major role for AQP5 in dynamic fine-tuning of the intracellular H_2_O_2_ concentration, and consequently in activating signaling networks related to cell survival and cancer progression, highlighting AQP5 as a promising drug target for cancer therapies.

## 1. Introduction

Aquaporins, which are expressed in almost every organism and tissue, constitute a highly conserved group of transmembrane proteins that are crucial for cell homeostasis and volume regulation. AQPs are assembled in a homotetrameric structure in membranes, each monomer being a functional channel that facilitates a rapid bidirectional flux of water, and, in some cases, small uncharged solutes like glycerol, in response to osmotic or solute gradients [1]. The thirteen human isoforms (AQP0–AQP12) are expressed in a cell- and tissue-dependent manner, and are subdivided according to their selectivity and sequence homology. Classical or orthodox aquaporins are considered mainly selective to water (AQP0, AQP1, AQP2, AQP4, AQP5, AQP6, AQP8), while aquaglyceroporins also mediate glycerol and urea fluxes (AQP3, AQP7, AQP9, AQP10), and super aquaporins show lower homology and are found in subcellular membranes (AQP11, AQP12). Among these AQPs, some isoforms have also been shown to permeate ammonia [1,2]. More recently, a fourth sub-group comprising isoforms that facilitate the diffusion of H_2_O_2_ through cell membranes has been identified and named peroxiporins [3,4,5,6,7].

The importance of AQPs for water homeostasis and energy metabolism has been extensively reported [8,9]. More recently, a growing number of studies have reported the involvement of AQPs in cell proliferation, angiogenesis, and migration [10,11], and cellular resistance to oxidative stress [6]. It is well known that high intracellular levels of ROS, unbalancing the cellular redox state, play a key role in tumorigenesis, either by damage of proteins, lipids, and nucleic acids or by abnormal induction of signaling pathways [12]. However, ROS also act as signaling molecules regulating a number of physiological processes [13]. H_2_O_2_ is a small molecule produced by aerobic metabolism or in response to extracellular insults, being the main ROS participating in oxidative sensing and signaling [14]. Depending on its intracellular concentration, H_2_O_2_ can have a dual effect [15]. In this context, facilitated diffusion of H_2_O_2_ across cell membranes may play a crucial role in the fine-tuning of cell oxidative status and its biological consequences.

Several studies have reported the overexpression of AQPs in human tumors of different origin, frequently correlated with tumor stage and aggressiveness [11], unveiling a putative association with their role as peroxiporins [5,16,17,18]. In particular, AQP3 and AQP5 were found to be aberrantly expressed in a variety of human tumors, and have been considered potential therapeutic targets and biomarkers with prognostic value (for a recent review, see Reference [19]). The expression level of AQP3 was found to correlate with cancer cell migration through a mechanism involving its hydrogen peroxide transport function [4,18].

In a previous study, we showed that rat AQP5 also mediates H_2_O_2_ membrane diffusion and is involved in oxidative cell response [6]. In addition, the transport activity of phosphorylated rAQP5 could be regulated by acidification, a condition that is favored in cancer tissues. We thus hypothesized that, in addition to water, human AQP5 may also channel H_2_O_2_, and that the high level of expression and peroxiporin activity may play a key role in cellular adaption to oxidative stress, with impact on cancer cell migration. In the present study, we expressed human AQP5 in a yeast cell model, and first investigated its functional regulation by phosphorylation and pH, disclosing the important residues for water permeation as well as the ability of AQP5 to facilitate H_2_O_2_ diffusion across cell membranes. Secondly, we evaluated the contribution of AQP5 to cell survival under oxidative conditions and to cell adaptation to an oxidative environment. Finally, we measured the H_2_O_2_ influx rate mediated by AQP5 in human pancreatic cancer cells, and revealed its impact on cell migration under oxidative stress.

We found that AQP5 expressed in yeast localizes to the plasma membrane and increases the membrane water permeability 10-fold compared to the control. Investigation of residues important for functional regulation revealed His173, localized in the selectivity filter, as crucial for channel permeability, independent of pH. Phosphorylation of Ser183 impaired AQP5 function, probably due to its proximity to His173, interacting with the negative charge of the phosphate group. Importantly, we showed for the first time that human AQP5 transports H_2_O_2_ and modulates cell resistance to oxidative stress. Moreover, in human pancreatic cancer cells, the measured AQP5-mediated H_2_O_2_ influx rate indicates a highly efficient peroxiporin activity. Cell migration was similarly suppressed by AQP3 or AQP5 gene silencing, and was promoted by AQP-facilitated H_2_O_2_ transport. Altogether, these results unveil a major role of AQP5 in cancer progression, highlighting its potential as a drug target for cancer therapies.

## 2. Results

### 2.1. Human AQP5 is Localized and Functional at the Yeast Plasma Membrane

**Figure 1:**
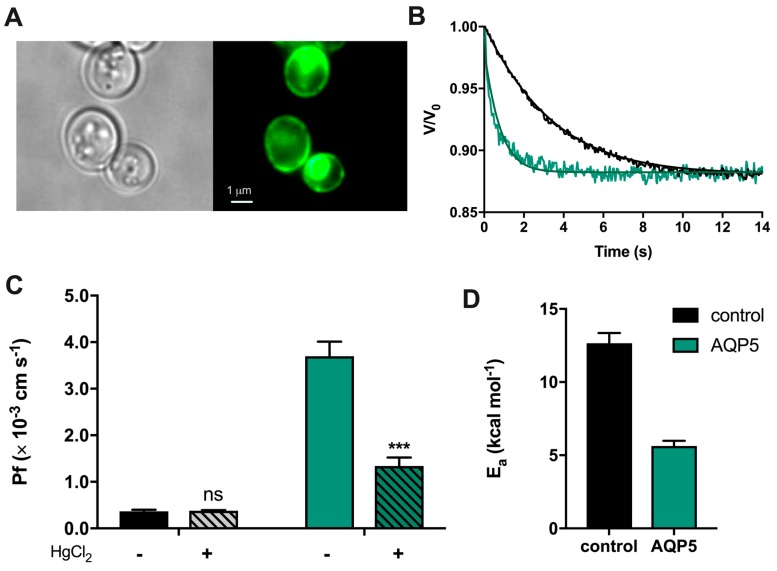
Expression and function of human aquaporin-5 (AQP5) in transformed *Saccharomyces cerevisiae* cells. (**A**) Phase contrast (left) and epifluorescence (right) microscopy images of yeast aqy-null cells transformed with GFP-tagged human AQP5 (100× objective). (**B**) Representative time course of relative cell volume (V/V_0_) changes after a hyperosmotic shock inducing cell shrinkage. (**C**) Water permeability coefficients of control and cells expressing human AQP5 before and after HgCl_2_ incubation for 5 min at room temperature measured at 23 °C and pH 5.1. Data are shown as mean ± SEM of 10 measurements. (**D**) Activation energy (E_a_) for water permeation of control and AQP5 cells. Data are shown as mean ± SEM of three independent experiments. Significance levels: ns, non-significant; *** *p* < 0.001.

To evaluate human AQP5 function, yeast cells depleted of endogenous aquaporins (aqy-null) were transformed with either the empty plasmid pUG35 (control cells) or the plasmid encoding hAQP5. Prior to functional analysis, expression and localization of AQP5 at the yeast plasma membrane were verified by fluorescence microscopy using GFP-tagging (Figure 1A). AQP5 function was evaluated by stopped-flow fluorescence. Cells were loaded with the volume-sensitive dye carboxyfluorescein and exposed to a hyperosmotic solution with an impermeant solute, inducing cell shrinkage. Water permeability was evaluated by monitoring the time course of fluorescence output that reflects the transient volume change. As shown in Figure 1B, when exposed to a hyperosmotic solution, cells expressing AQP5 readjust their final volume and reach their new osmotic equilibrium faster than control cells, evidencing water channeling. The water permeability coefficient (P_f_) was 10-fold higher for AQP5-transformed yeast cells ((3.70 ± 0.31) × 10^−3^ cm s^−1^ and (0.37 ± 0.03) × 10^−3^ cm s^−1^ for AQP5 and control, respectively). Incubation with 0.5 mM HgCl_2_ markedly reduced water permeability of AQP5-transformed yeast cells (≈64%) without affecting control cells (Figure 1C), indicating that AQP5 is a mercury-sensitive water channel. Finally, the activation energy for water transport (E_a_), which distinguishes passive water diffusion through lipid bilayer from AQP-mediated diffusion, was lower for AQP5 cells (5.63 ± 0.36 kcal mol^−1)^ compared to the control (12.66 ± 0.69 kcal mol^−1^) (Figure 1D). These results show that AQP5 is assembled into a functional water channel in yeast.

### 2.2. S183 and H173 are Important Residues for AQP5 Gating

Recent evidence supports the idea that human AQPs can be gated via different mechanisms, including pH and phosphorylation [20,21]. Regarding AQP5, regulation was proposed to involve phosphorylation at Ser156 in cytoplasmic loop D to rapidly and reversibly regulate AQP5 plasma membrane abundance [22]. Phosphorylation of AQP5 in its PKA consensus site (S156) induced colon cancer cell proliferation via the Ras/ERK/Rb pathway [23]. In addition, in silico studies suggested a second gating mechanism [24] where the AQP5 monomer undergoes conformational changes varying between an open/close state and wide/narrow state. The authors proposed that the AQP5 channel could change from open to closed by a tap-like mechanism at the cytoplasmic end, induced by translation of the His67 side chain inside the pore, blocking the water passage, and that the selectivity filter (SF) regulates the rate of water flux when the channel is open. In this case, AQP5 channels could exhibit two different conformations (wide and narrow), determined by the proximity of the H173 side chain to S183: when these residues get close (<5.5 Å), the SF turns to the narrow conformation and water passage is restricted. The channel constriction induced by H173 side chain orientation determines the two states, wide/narrow, when the cytoplasmic end gate switches from closed to the open state. In addition, our recent study with rAQP5 indicated that channel widening results from deprotonation when the protein is in the phosphorylated state [6]. Thus, using the same yeast system, here we investigated mechanisms of human AQP5 gating by phosphorylation and pH.

**Figure 2:**
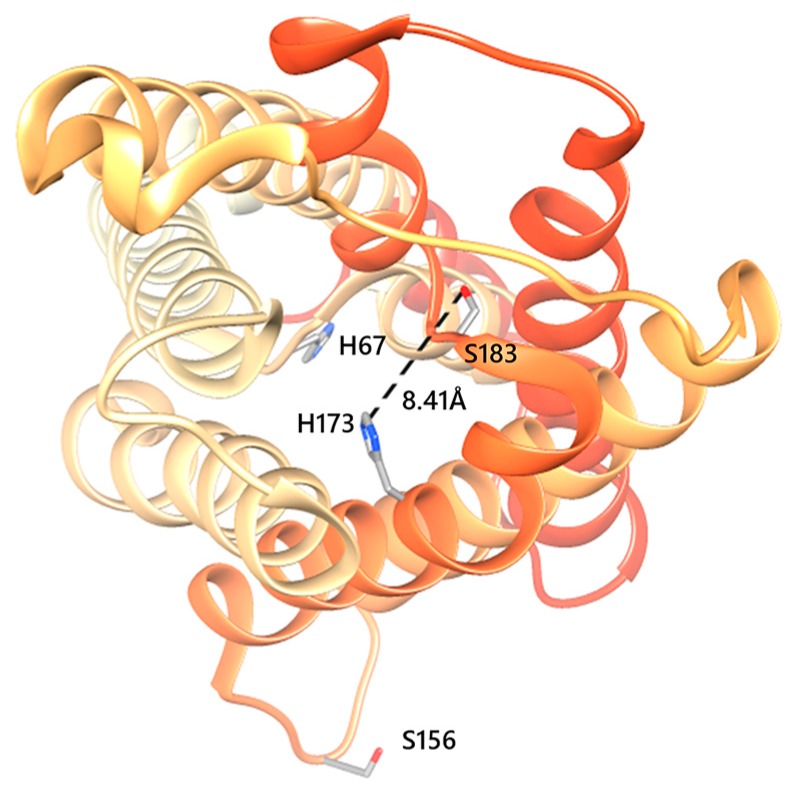
Structure of human AQP5 monomer. Top view of AQP5 monomer with phosphorylation consensus sites Ser156 localized in intracellular loop D, and Ser183 localized in the selectivity filter along with His173. As proposed, when His67 side chain rotates outside the pore, it allows water passage through the pore (open state) [24]. In such cases, if the proximity of His173 to Ser183 (D1) is 7Å<D1<10Å, the AQP5 monomer shows a wide conformation. Structures were generated with Chimera (http://www.cgl.ucsf.edu/chimera) and are based on the AQP5 X-ray structure (PDB databank code 3D9S).

**Figure 3:**
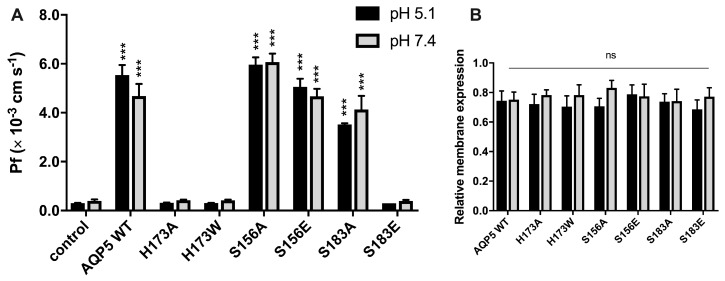
Residues involved in water permeability through AQP5. (**A**) Water permeability coefficients of control and cells expressing AQP5 WT and mutants, performed at 23 °C and pH 5.1 and 7.4. Data are shown as mean ± SEM of 10 measurements; (**B**) Relative membrane expression of AQP5 WT and mutants at pH 5.1 and pH 7.4, calculated from fluorescence intensity profiles (10 cells in each experimental condition, 4 profiles for each cell, from at least 3 independent experiments). Significance levels: ns, non significant, *** *p* < 0.001, compared to control.

We generated point mutations in the AQP5 aromatic/arginine region and in intracellular loop D (Figure 2). Mutations to change wide and narrow state were obtained by substitution of histidine (H) 173 with alanine (A) and with tryptophan (W), respectively. Mutations preventing phosphorylation of S156 and S183 were obtained by substitution of serine (S) with alanine (A). Mutations to mimicking the charge state of AQP5 phosphorylated at the same serine residues were performed by substitution of serine (S) with glutamic acid (E). Water permeability of yeast cells expressing wild-type AQP5 (WT) or AQP5 mutants was determined at 23 °C at both pH 5.1 and pH 7.4 (Figure 3A). Expression and localization of all AQP5 mutants was confirmed at pH 5.1 and pH 7.4 by fluorescence microscopy using GFP-tagging (Figure S1). All yeast clones displayed similar GFP-fluorescence intensity at the plasma membrane (Figure S1 and Figure 3B), indicating that the observed differences in permeability cannot be assigned to impairment of AQP5 trafficking due to mutations.

Permeability experiments with AQP5 WT, mutants and control cells performed at two pHs (pH 5.1 and 7.4), showed that alteration of external pH did not affect water permeability, and that this is not dependent on protein phosphorylation. A recent study reported that phosphomimetic mutation of S156E increased membrane expression of AQP5 in HEK293 cells [22]. In our work, preventing or mimicking S156 phosphorylation did not alter AQP5 membrane expression (Figure 3B), nor did it modify AQP5 activity at any pH tested, which may in part be explained by a different signaling pathway for AQP5 trafficking used by yeasts. However, water permeability was fully blocked when histidine was mutated (H173A and H173W). Histidine is a highly conserved residue in the selective filter of water-specific aquaporins, and is considered crucial for selectivity and transport [25]. The observed impairment of water permeability by H173 mutation (Figure 3A) validates the previously reported in silico data.

To experimentally investigate the phosphorylation gating mechanism previously proposed in silico [24], we measured the water permeability of AQP5-S183A and AQP5-S183E mutant yeast cells. Impairment of S183 phosphorylation by mutation to alanine (S183A) did not affect water permeability, suggesting that the AQP5 channel remains in a wide conformation, probably because the distance between the H173 side chain and A183 is above 5.5 Å. However, the phosphomimetic mutation of S183 to glutamate (S183E) impaired permeability, indicating that the proximity of S183 to H173, mainly supported by the negative charge of the phosphate group, is responsible for pore constriction. This new AQP5 gating mechanism, involving phosphorylation of S183 at the selectivity filter (SF), has never been reported.

### 2.3. Human AQP5 Transports Hydrogen Peroxide

Several studies have reported H_2_O_2_ transport by a few human AQP isoforms, including AQP3 [4], AQP8 [3,26], and AQP9 [7]). Regarding AQP5, our group revealed that the rat isoform could also mediate H_2_O_2_ transport [6]. Sequence alignment of human and rat AQP5 isoforms reveals 91% sequence identity, and led us to investigate if human AQP5 can facilitate H_2_O_2_ permeation through membranes.

**Figure 4:**
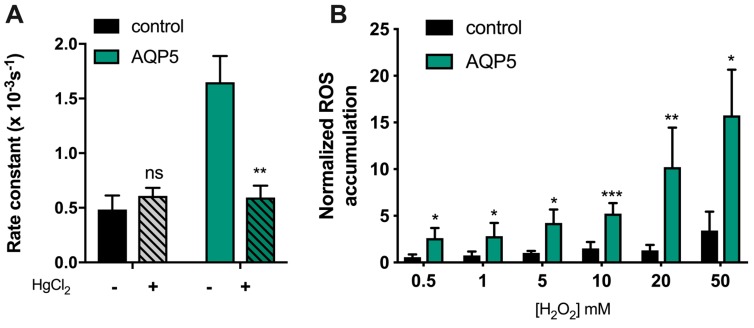
AQP5-mediated H_2_O_2_ uptake. (**A**) First-order kinetic rate constant (s^−1^) of H_2_O_2_ consumption of yeast cells measured with the Clark electrode (O_2_ measurement), before and after incubation with 0.5 mM HgCl_2_ for 5 min at RT. (**B**) AQP5-facilitated H_2_O_2_ consumption of yeast cells is concentration-dependent. Cells were incubated with a range of H_2_O_2_ concentration from 0.5 mM to 50 mM and ROS accumulation was recorded over 60 min, following H_2_-DCFDA fluorescence signal. Rate constant of ROS accumulation without H_2_O_2_ for each yeast strain was used to normalized rate constant of ROS accumulation. Values are shown as mean ± SEM of at least 3 independent experiments. Significance levels: ns, non-significant, * *p* < 0.05, ** *p* < 0.01, *** *p* < 0.001.

Thus, we measured the consumption of external hydrogen peroxide by yeast cells expressing human AQP5 using an electrochemical assay (O_2_ electrode). Briefly, hydrogen peroxide was added to cell suspensions, and H_2_O_2_ uptake was evaluated by monitoring its conversion into O_2_ with a Clark electrode after addition of catalase in excess to samples of the cell suspension. Since we found that human AQP5 is not pH-regulated, we performed the assays at a pH optimal for yeast cells (pH 5.1). As depicted in Figure 4A, the rate constant of H_2_O_2_ consumption was three-fold higher for yeast cells expressing AQP5 compared to control cells. In addition, incubation with 0.5 mM HgCl_2_, shown above to inhibit AQP5 water permeability, reduced H_2_O_2_ consumption to the basal level corresponding to diffusion through the lipid bilayer, not affecting control cells. These data confirm that AQP5 can facilitate H_2_O_2_ membrane permeability. Since H_2_O_2_ uptake measured by the Clark electrode relays on its extracellular disappearance, and to assure that the high rate of H_2_O_2_ consumption observed for AQP5-yeast cells was due to cellular uptake, we loaded the cells with the ROS sensitive fluorescence probe H_2_-DFCDA and followed the intracellular increase in fluorescence after incubation with a range of H_2_O_2_ concentrations. As depicted in Figure 4B, the rate of ROS accumulation was dependent on H_2_O_2_ concentration, largely facilitated by AQP5 expression.

### 2.4. AQP5 Regulates Cellular Resistance to Oxidative Stress

The involvement of ROS in initiation, promotion, and progression of cancer and their effect on cell behavior depends on concentration, time of exposure, and cellular antioxidant defense, among other factors [27]. In fact, ROS can contribute to the regulation of cell fate, including anti-cancer actions (e.g., by promoting senescence, apoptosis, necrosis, or other types of cell death, and inhibiting angiogenesis) or pro-cancer actions (promoting proliferation, invasiveness, angiogenesis, metastasis, and suppressing apoptosis) [27]. Recent evidence showed a novel role for AQP3-mediated H_2_O_2_ transport in the mechanism of breast cancer cell migration [18]. Therefore, we aimed to investigate the physiological role of AQP5-mediated H_2_O_2_ transport in cell resistance and growth.

**Figure 5:**
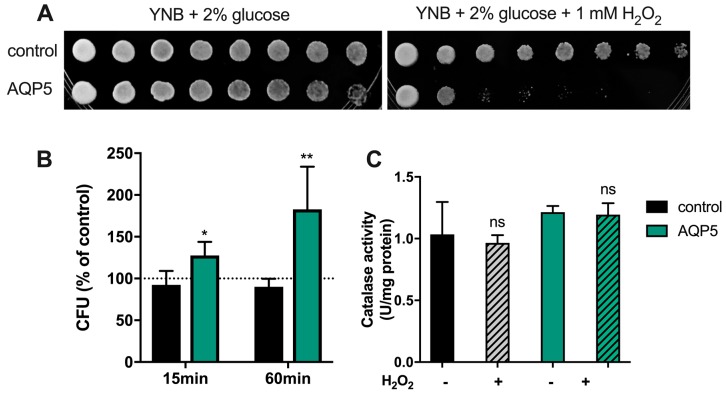
Yeast cell sensitivity assay and catalase activity under oxidative stress. (**A**) Growth assay of yeast cells on medium containing H_2_O_2_. Yeast suspensions were spotted at 10-fold dilutions on solid YNB plates without or with 1mM H_2_O_2_. Growth was recorded after two days at 28 °C. Photographs shown are representative of three independent experiments with consistent results. (**B**) Survival test of yeast cells in liquid culture after treatment with 1 mM H_2_O_2_ for 15 and 60 min at 30 °C. Survival is expressed by the percentage of colony forming units (CFU) relative to untreated controls. Significance levels: * *p* < 0.05 and *p* < 0.01 vs. control. (**C**) Catalase activity before and after treatment with 1 mM H_2_O_2_ for 60 min at 30 °C. Values are mean ± SEM of triplicates. Significance level: ns, non-significant vs. non-treated cells.

First, we evaluated the effect of long exposure to oxidative stress (Figure 5A). Yeast cells were grown on solid medium containing 1 mM H_2_O_2_ for two days, and it could be observed that cells expressing AQP5 were much more sensitive to oxidative stress than control cells, which were only slightly affected.

Subsequently, cells were exposed to short-term oxidative stress, i.e., in the presence of 1 mM H_2_O_2_ for 15 and 60 min (Figure 5B). Results indicate that, when exposed to H_2_O_2_, cells expressing AQP5 can not only survive to oxidative stress, but also grow better (1.5- and 2-fold after 15 and 60 min, respectively, compared to non-treated cells). This “overcoming” effect was not observed in control cells that were depleted of endogenous aquaporins, in which H_2_O_2_ slowly diffused through membrane lipids. As a control, catalase activity was also evaluated in non-treated cells and after incubation with 1 mM H_2_O_2_ for 60 min; absence of statistical differences indicate that this antioxidant scavenger is not responsible for the observed cellular resistance of AQP5-yeast cells.

### 2.5. AQP5 Shows High Peroxiporin Activity in Pancreatic Cancer Cells

**Figure 6:**
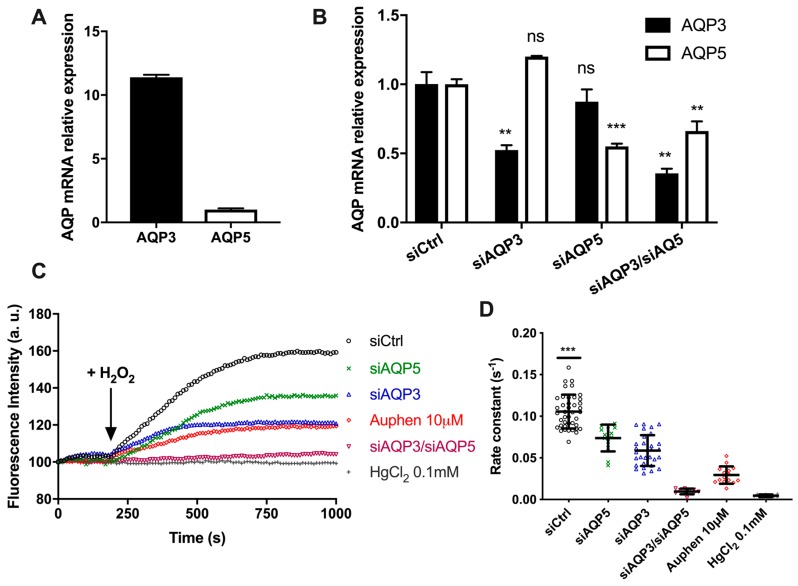
AQP5-mediated H_2_O_2_ uptake in pancreatic cancer cells. (**A**) AQP3 and AQP5 mRNA expression levels in BxPC3 cells using β-actin as a reference; (**B**) AQP3 and AQP5 mRNA expression levels in BxPC3 cells transiently transfected with siAQP3 and siAQP5. Data represent mean ± SEM from three independent experiments. Significance levels: ns, non-significant, ** *p* <0,01, *** *p* < 0.001 vs. siCtrl; (**C**) Representative traces of H_2_-DCFDA fluorescence increase after addition of 100 µM H_2_O_2_; (**D**) First-order kinetic rate constant (s^−1^) of H_2_O_2_ influx through endogenous AQP3 and AQP5 expressed in BxPC3 cells after addition of 100 µM H_2_O_2._ Data shown are individual values and mean ± SEM of three independent experiments. Significance levels: *** *p* < 0.001 vs. siAQP5, siAQP3, siAQP5, siAQP3/siAQP5, Auphen 10 µM and HgCl_2_ 0.1 mM.

The BxPC3 pancreatic cancer cell line was tested to confirm the yeast results and further investigate the biological relevance of AQP-mediated H_2_O_2_ transport. Evaluation of relative gene expression by quantitative real-time RT-PCR demonstrated that both AQP3 and AQP5 were expressed in BxPC3, although with different levels of expression (Figure 6A).

Since both AQP5 and AQP3 transport H_2_O_2_, and to distinguish the contribution of each isoform to H_2_O_2_ permeability, we used BxPC3 cells silenced for AQP3, for AQP5, or double silenced for AQP3–AQP5 to evaluate H_2_O_2_ permeability. The knockdown efficiency was evaluated by RT-qPCR after 48 h and compared with the non-targeting control construct, showing a reduction of AQP3 and AQP5 expression of around 50% (Figure 6B).

H_2_O_2_ membrane transport was evaluated by epifluorescence microscopy. The rate of fluorescence increase of individual cells incubated with 10 µM H_2_-DCFDA was monitored before and after addition of 100 µM H_2_O_2_ (Figure 6C). As depicted, the rate of H_2_O_2_ uptake was maximal for control cells that expressed both AQP3 and AQP5. Cells silenced for AQP5 (siAQP5) still expressing AQP3, and cells silenced for AQP3 (siAQP3) still expressing AQP5, responded to H_2_O_2_ addition with a similar rate of fluorescence increase, approximately half the control (Figure 6D). In AQP3–AQP5 silenced cells, H_2_O_2_ uptake was almost abolished, and a similar effect was seen when cells were treated with the non-specific aquaporin inhibitor HgCl_2_. Moreover, when control cells were incubated with the AQP3 inhibitor Auphen [28], inhibiting AQP3 but not AQP5, the rate of H_2_O_2_ uptake was considerably decreased but still significant, due to AQP5 peroxiporin activity.

Interestingly, the level of AQP3 expression in BxPC3 cells was 11-fold higher than AQP5, and the silencing was equally efficient for both AQPs. However, the measured rate of H_2_O_2_ permeation was similar for AQP3 and AQP5, suggesting that AQP5 may have a higher capacity for H_2_O_2_ flux, resulting in a highly efficient peroxiporin.

### 2.6. Effect of AQP-Mediated H_2_O_2_ Transport on Cell Migration

To investigate whether AQP5-mediated H_2_O_2_ transport underlies signal transduction in pancreatic cancer progression, we evaluated the rate of cell migration before and upon cell treatment with extracellular H_2_O_2_ of BxPC3 cells silenced for AQP3 (siAQP3), for AQP5 (siAQP5), and control cells transfected with a non-targeting construct (siCtrl).

**Figure 7:**
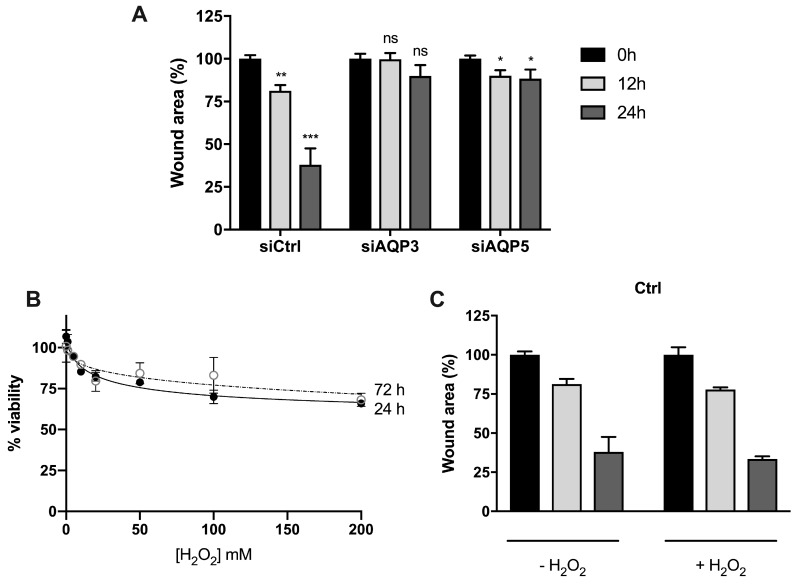

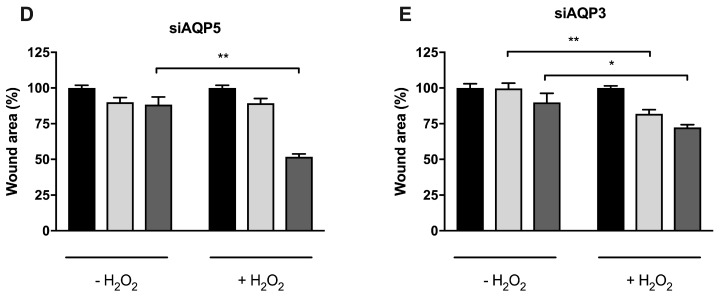
Cell migration of BxPC3 cells upon oxidative stress. (**A**) Cell migration of cells transiently transfected with corresponding siRNA (control, AQP3, and AQP5) in normal media. Significance levels: ns, non-significant, * *p* < 0.05, ** *p* < 0.01, *** *p* < 0.001 vs. time 0. (**B**) Cell viability determined by MTT assay after cell exposure to a range of H_2_O_2_ concentrations for 24 h and 72 h. (**C**) Cell migration of control cells, (**D**) cells silenced for AQP5, and (**E**) cells silenced for AQP3, before and after addition of 100 µM H_2_O_2_. Wound repair was monitored at 12 h and 24 h under light microscopy, and cell migration was normalized to original wound area at time 0. Results are mean ± SEM of triplicate experiments. Significance levels: * *p* < 0.05, ** *p* < 0.01 vs. non-treated cells.

Studies on monolayers of BxPC3 control cells (siCtrl) showed a partial recovery of wound area after 12 h (around 20%; *p* < 0.01) and significant recovery after 24 h (around 60%; *p* < 0.001) (Figure 7A). However, silencing AQP3 completely impaired cell migration, and silencing AQP5 substantially reduced cell migration and wound closure compared to control cells. These results are consistent with previous reports in AQP3-null mice, breast cancer, and sarcoma cells [18,29,30], and indicate that AQP3 and AQP5 play a role in the regulation of cell migration in pancreatic cancer cells, representing a promising target for the treatment of human pancreatic adenocarcinoma.

Previous to the oxidative migration assay, we assessed the effect of external H_2_O_2_ stimulus on the viability of BxPC3 cells. As depicted, the addition of H_2_O_2_ up to 200 µM induced a maximal 30% loss of cell viability (Figure 7B). Thus, in subsequent experiments, we used 100 µM H_2_O_2_, assuring >70% cell viability for migration assays.

The contribution of each AQP to cell migration under oxidative stress was investigated in control cells (Figure 7B), in siAQP3 (Figure 7D), or in siAQP5 cells (Figure 7E) before and after treatment with 100 µM H_2_O_2_. Downregulation of AQP5 or AQP3 gene expression had a strong impact on cell migration of non-treated cells, which, even at 24 h, were not able to decrease the wound area when compared to control cells (Figure 7D,E). However, in siAQP5 cells that still express AQP3, oxidative treatment induced a significant recovery at 24 h (50% wound area), similar to control cells, possibly due to H_2_O_2_ diffusion via AQP3, as previously reported for breast cancer cells [18]. When siAQP3 cells were treated with H_2_O_2_, the wound recovery was detected earlier at 12 h, although to a lesser extent, showing the positive effect of H_2_O_2_ diffusion via AQP5 on cell migration. These data demonstrate for the first time that AQP5 expression and peroxiporin activity in pancreatic cancer cells is critical for cell migration and tumor spread.

## 3. Discussion

In biological systems, ROS are generated endogenously by mitochondrial respiratory chain and oxidase enzymes, or in response to extracellular stimuli. ROS products, including H_2_O_2_, contribute to oxidative stress and can lead to initiation and progression of several chronic diseases, like atherosclerosis, diabetes, neurodegeneration, and tumorigenesis. However, at low concentration, ROS can regulate signaling pathways and physiological processes, including cell growth, differentiation, and migration. Recent studies showed that some mammalian AQPs can channel H_2_O_2_ across the cell plasma membrane, and reported their involvement in signaling cascades and tumorigenesis [19]. AQP3-mediated H_2_O_2_ transport has been linked to cancer cell migration, explaining its overexpression in cancer tissues. Rat AQP5 has been characterized as peroxiporin, but studies with the human isoform were lacking. Knowing that AQP5 is also highly expressed in human tumors [16], we investigated AQP5 functional regulation and its ability to transport H_2_O_2_ that may account for a role in tumor progression.

Here, we report that human AQP5 facilitates H_2_O_2_ uptake in hAQP5-transformed yeast cells, an ability also detected in a cultured pancreatic adenocarcinoma cell line. It is worth mentioning that AQP5 was found to be overexpressed in pancreatic adenocarcinoma biopsies of patients compared with matched normal pancreas tissues, being correlated with tumor stage and aggressiveness [17].

We found that AQP5 expression increases yeast sensitivity to oxidative damage after a long-term insult (48 h), but renders cells more resistant to short-term oxidative stress, evidencing the positive contribution of AQP5-mediated H_2_O_2_ diffusion to cell growth and survival. Mutagenesis studies demonstrated that while phosphorylation of S156 at the cytoplasmic end does not affect permeability, residue His173 located in the selective filter is crucial for water permeability and possibly interacts with phosphorylated S183 for permeability regulation, resulting in blockage of the pore. This mechanism of gating might be involved in the fine-tuning of cell sensitivity/resistance to oxidative external conditions, where NOX-produced H_2_O_2_ is taken up via AQPs triggering signaling cascades and induces cell proliferation and migration. In fact, both AQP5 and AQP3 showed measurable peroxiporin activity in pancreatic cancer cells, with AQP5 showing higher efficiency than AQP3. Moreover, both AQP5 and AQP3 were revealed to be crucial for cell migration, as shown here for BxPC3 cells, for which cell migration was drastically reduced when these peroxiporins were silenced.

The recovery of the migration rate by external oxidative stimulus demonstrates that when AQP3 or AQP5 are downregulated, signaling events triggered by H_2_O_2_ are blocked by the permeability barrier imposed by biomembranes, and additional H_2_O_2_ is needed to force cell migration. It has been reported that during normal conditions (eustress) the gradient between extracellular and intracellular H_2_O_2_ is higher than 200-fold [31,32,33]; the presence of aquaporins will decrease this gradient, favoring H_2_O_2_ permeability and prompting cellular processes like proliferation and migration [15]. The observation that silenced-AQP3 or -AQP5 cells recover cell migration rate by treatment with H_2_O_2_ perfectly agrees with this notion.

Altogether, our findings demonstrate that AQP5 can play an important role in cancer cell survival. By allowing a dynamic fine-tuning of intracellular H_2_O_2_ to activate signaling networks related to cell survival and proliferation, AQP5 can regulate cellular resistance to oxidative stress as well as facilitate cancer cell migration, and represents a promising target for the development of cancer therapies.

## 4. Materials and Methods

### 4.1. Yeast Strains and Growth Conditions

Transformed yeast strain was grown in YNB medium (2% *w*/*v* glucose, 0.67% (DIFCO) yeast nitrogen base) supplemented with the adequate requirements for prototrophic growth [34] and maintained in the same medium with 2% (w/v) agar. For all experiments, the same medium was used for yeast cell growth to mid exponential phase (OD_600_ 1.0).

### 4.2. Cloning and Heterologous Expression of AQP5 in S. Cerevisiae

Two sets of expression plasmids were generated. One set expresses non-tagged AQP5, while the other set expresses AQP5 C-terminally tagged with yeGFP. All plasmids were constructed by homologous recombination in yeast strain YSH1770 by co-transformation of AQP5 derived PCR fragments and *Bam*HI, *Sal*I, *Hin*dIII digested pUG35, as described before [35]. Primers used are shown in Table 1. The nucleotide sequence of all constructs was verified by DNA sequencing at Eurofins Genomics, Germany.

### 4.3. AQP5 Subcellular Localization by Fluorescence Microscopy

For subcellular localization of GFP-tagged AQP5 in *S. cerevisiae*, yeast cells in the mid-exponential phase were observed using a Zeiss Axiovert 200 fluorescence microscope, at 495 nm excitation and 535 nm emission wavelengths. Fluorescence microscopy images were captured with a digital camera (CoolSNAP EZ, Photometrics, Huntington Beach, CA, USA) using the Metafluor software (Molecular Devices, Sunyvale, CA, USA).

### 4.4. Cell Culture

Biopsy xenograft of Pancreatic Carcinoma line-3 (BxPC3) was obtained from ATCC (catalog no. CRL-1687) and cultured at 37 °C in 5% CO_2_. Cells were grown in RPMI1640 medium with 10% FBS and 1% penicillin/streptomycin. Medium was changed every 2–3 days and experiments were performed with 70% to 80% cell confluence.

### 4.5. Transfection with siRNA for AQP Silencing

Short interfering RNA (siRNA) targeting human AQP3 (ID: s1521) and human AQP5 (ID: s1527) were purchased from Ambion. Silencer^®^ Negative Control siRNA #1 (Ambion, ThermoFisher Scientific, Waltham, MA, USA) was employed as the negative control to ensure silencing specificity in all the experiments. Briefly, medium was removed and cells were supplied with Opti-MEM I reduced serum medium without antibiotics (Opti-MEM) (Life technologies, ThermoFisher Scientific, Waltham, MA, USA). siRNA (30 pmol) were diluted in Opti-MEM and mixed with Lipofectamine™ RNAiMAX transfection reagent (Invitrogen, ThermoFisher Scientific, Waltham, MA, USA) pre-diluted in Opti-MEM according to the manufacturer’s instructions. After 5 min incubation at room temperature, the mix was added to the cells and incubated at 37 °C in 5% CO_2_. For double silencing, both AQP3-RNAiMAX and AQP5-RNAiMAX complexes were added to the cells. After 48 h of incubation, the knockdown efficiency was evaluated by quantitative real-time RT-PCR, as below.

### 4.6. RNA Isolation and Real Time RT-PCR

Total RNA was extracted from cultured cells using TRIzol Reagent (Invitrogen), according to the manufacturer’s protocol. The RNA was treated with RNase-free DNase I (Sigma-Aldrich, St. Louis, MO, USA) to avoid contamination with genomic DNA. Extracted RNA was quantified with Nanodrop™ 2000c spectrophotometer. For template cDNA synthesis, 1 µg of total RNA was reverse transcribed in a 20 µL final volume using random hexamers primers (Roche Applied Science, Penzberg, Germany) and 200 units of M-MLV reverse transcriptase (Invitrogen), as previously described [36].

Real-time PCR reactions were carried out using a CFX96 Real-Time System C1000 (BioRad, Hercules, CA, USA), the TaqMan Universal PCR Master Mix (Applied Biosystems Thermo Fisher Scientific, Waltham, MA, USA), and the following specific TaqMan pre-designed gene expression primers: AQP1 (Hs01028916_m1), AQP3 (Hs01105469_g1), AQP5 (Hs00387048_m1), and ACTB (Hs99999903_m1) (Applied Biosystems, ThermoFisher Scientific, Waltham, MA, USA). The cDNA was amplified at the following conditions: 50 °C for 2 min, 95 °C for 10 min, followed by 40 cycles of 15 s at 95 °C and 1 min at 60 °C.

The relative quantification of gene expression was determined using the 2^–ΔCt^ method (adapted from Reference [37]). Using this method, we obtained the fold variation in AQP gene expression normalized to an endogenous control (β-actin).

### 4.7. Migration Assay

BxPC3 cells were seeded in six well microplates at a density of 0.15 × 10^6^ cells/well and were allowed to adhere for 24 h prior to AQP silencing. After 48 h incubation with silencing reagent, a wound was made with an even trace in the middle of the monolayer using a sterile 10 µL pipette tip. After washing three times with phosphate buffered saline (PBS) to remove cell debris, cells were incubated with vehicle or with 100 µM H_2_O_2_ prepared in low serum (2% FBS) RPMI medium (Life technologies, ThermoFisher Scientific, Waltham, MA, USA). The cells were then incubated at 37 °C in a 5% CO_2_ incubator, and images of the wound were captured at intervals of 2 h. The distance of the wound was measured under a light microscope and analyzed using the software ImageJ (https://imagej.net). Wound closure was normalized to original wound area at time 0. All samples were tested in triplicate, and the data are expressed as the mean ± SEM.

### 4.8. Water Permeability Measurements

For water permeability assays, yeast transformants grown up to OD_600_ 1.0 were harvested by centrifugation (4000× *g*; 10 min; 4 °C), washed three times and resuspended in ice cold sorbitol (1.4 M) K+-citrate (50 mM pH 5.1 or 7.4) buffer up to a concentration of 0.3 g mL^−1^ wet weight, and cells were incubated on ice for at least 90 min. Prior to permeability assays, cells were preloaded with the non-fluorescent precursor 5-(and-6)-carboxyfluorescein diacetate (CFDA, 1 mM, 10 min at 30 °C), which is intracellularly hydrolyzed, yielding the impermeable fluorescent form (CF). Cells were then diluted (1:10) in 1.4 M sorbitol buffer and immediately used for experiments.

Permeability assays were performed by stopped-flow fluorescence spectroscopy, as previously described [38], using a HI-TECH Scientific PQ/SF-53 stopped-flow apparatus, which has a 2 ms dead time, at a controlled temperature, interfaced with a microcomputer. Experiments were performed at temperatures ranging from 9 to 34 °C. Four runs were usually stored and analyzed at each experimental condition. In each run, 0.1 mL of cell suspension was mixed with an equal volume of hyperosmotic sorbitol buffer (2.1 M sorbitol, 50 mM K-citrate, pH 5.1 or 7.4) producing an inwardly directed gradient of the impermeant sorbitol solute that induces water outflow and cell shrinkage. Fluorescence was excited using a 470 nm interference filter and detected using a 530 nm cut-off filter. The time course of cell volume change was followed by fluorescence quenching of the entrapped fluorophore (CF). The initial rate constant of volume changes (k) was obtained by fitting the time course of fluorescence to a one phase exponential. The osmotic water permeability coefficient, Pf, was estimated from the linear relationship between Pf and k [38], Pf = k(Vo/A)(1/Vw(osmout)), where Vw is the molar volume of water, Vo/A is the initial volume to area ratio of the cell population, and (osmout) is the final medium osmolarity after the osmotic shock. The osmolarity of each solution was determined from freezing point depression by a semi-micro-osmometer (Knauer GmbH, Germany). The activation energy (Ea) of water transport was evaluated from the slope of the Arrhenius plot (ln Pf as a function of 1/T) multiplied by the gas constant R.

### 4.9. Hydrogen Peroxide Consumption

The H_2_O_2_ consumption was measured in intact yeast cells. Cells were harvested by centrifugation (4000× *g*; 10 min at RT), resuspended in fresh growth media and incubated at 30 °C with orbital shaking. Hydrogen peroxide (50 µM) was added to intact yeast cells, and the consumption of H_2_O_2_ was measured in samples of the cell suspension by following O_2_ release with an oxygen electrode (Hansatech Instruments Ltd., Norfolk, UK) after the addition of catalase [39]. H_2_O_2_ consumption is reported as a first order rate constant (s^−1^) obtained from the slope of a semi-logarithmic plot of H_2_O_2_ concentration versus time.

### 4.10. Intracellular ROS Analysis

To evaluate H_2_O_2_ influx, oxidation kinetics of non-fluorescent probe 2′,7′-dichlorodihydrofluorescein diacetate (H_2_-DCFDA, Invitrogen, ThermoFisher Scientific, Waltham, MA, USA) to fluorescent 2′,7′-dichlorofluorescein was measured until 60 min from H_2_O_2_ administration, according to previously described methods [40].

Yeast transformants grown up to OD_600_ 1.0 were harvested by centrifugation (4000× *g*; 10 min; 4 °C) (Allegra^®^ 6 Series Centrifuges, Beckman Coulter^®^), washed three times with phosphate buffer 0.1 M pH 5 and resuspended in the same buffer to OD_600_ 1.4. Cells were then incubated with 5 µM H_2_-DCFDA for 45 min at 30 °C and washed one time with phosphate buffer 0.1 M. Cells were transferred to black multi-well plates and incubated with several concentrations of H_2_O_2_ (0.5–50 mM). Fluorescence intensity was measured after addition of H_2_O_2_ over time until 60 min in a microplate reader at an excitation/emission of 485/520 nm (FLUOstar Omega, BMG Labtech, Ortenberg, Germany). As control, H_2_O_2_ non-treated cells were incubated with 5 µM H_2_-DCFDA and fluorescence intensity was followed. The intracellular ROS accumulation was calculated from the slope of a plot of fluorescence intensity versus time and normalized to non-treated cells.

BxPC3 cells were seeded in six well microplates at a density of 0.15 × 10^6^ cells/well, and were allowed to adhere for 24 h prior to AQP silencing. After 48 h incubation with silencing reagent, H_2_O_2_ transport was measured in individual adherent cells on a coverslip. Briefly, cells were loaded with 10 µM H_2_-DCFDA for 30 min at 37 °C in 5% CO_2_. Next, cells were washed twice with Ringer Buffer (RB) pH 7.4 (140 mM NaCl, 2 mM CaCl_2_, 1 mM MgSO_4_, 1.5 mM K_2_HPO_4_, 10 mM glucose) and the coverslips with the cells were mounted in a closed perfusion chamber (Warner Instruments, Hamden, CT, USA) on the stage of a Zeiss Axiovert 200 inverted microscope, using a 40× epifluorescence oil immersion objective. Fluorescence was excited at wavelength 495/10 nm; emission fluorescence was collected with a 515/10 nm band pass filter. Data were recorded and analyzed using the Metafluor Software (Molecular Devices, Sunnyvale, CA, USA) connected to a CCD camera (Cool SnapTM EZ Photometrics, Tucson, AZ, USA). Cells were equilibrated in RB pH 7.4 for 2 min, and then 100 µM H_2_O_2_, freshly prepared in RB, was added directly to the cells. H_2_-DCFDA fluorescence was scanned every 10 s. For inhibition studies, cells were incubated with 10 µM Auphen for 15 min or 0.1 mM HgCl_2_ for 5 min at 37 °C in 5% CO_2_. H_2_O_2_ consumption is reported as a first order rate constant obtained from the slope of a semi-logarithmic plot of H_2_O_2_ concentration versus time.

### 4.11. Intracellular ROS Analysis

Qualitative growth assay was performed on solid YNB medium, supplemented with 2% (w/v) glucose, containing hydrogen peroxide. Solid YNB medium with 1 mM H_2_O_2_ was freshly prepared at the time of inoculation for oxidative stress experiments. Yeast strains were grown in liquid YNB medium, with orbital shaking, at 30 °C up to OD_600_ ≈ 1.0 corresponding to 1 × 10^7^ cells/mL. Cells were harvested by centrifugation (4000× *g*; 10 min; 24 °C) (Allegra^®^ 6 Series Centrifuges, Beckman Coulter^®^) and resuspended to OD_600_ 1.0 in fresh growth media and incubated with 50 µM curcumin or 50 µM naringenin at 30 °C with orbital shaking for 60 min. Cells were then harvested by centrifugation (4000× *g*; 10 min; 24 °C) (Allegra^®^ 6 Series Centrifuges, Beckman Coulter^®^) and resuspended to OD_600_ ≈ 10, and multi-well plates were prepared with serial 10-fold dilutions of the original concentrated culture up to 10^–8^; 3 µL suspensions were spotted with replica platter for 96 well plates device on plates containing YNB solid medium with and without H_2_O_2_ and incubated at 28 °C. Differences in growth phenotypes of yeast strains were recorded after 2 days of incubation.

Quantitative growth assay was performed on solid YPD medium. Yeast cells were grown overnight to mid exponential phase (OD_600_ 1.0). Cells were harvested by centrifugation (4000× *g*; 10 min; 24 °C) (Allegra^®^ 6 Series Centrifuges, Beckman Coulter^®^) and resuspended to OD_600_ 1.0 in fresh growth media and incubated with 1 mM H_2_O_2_ at 30 °C with orbital shaking for 15 and 60 min. Multi-well plates were prepared with serial 10-fold dilutions of each strain up to 10^−6^ and 3 µl suspensions were spotted on solid YPD plates. As a control for maximum viability, cells without treatment were also diluted and plated as described above. YPD plates were then incubated for 2 days at 28 °C until visible growth was observed and colonies were counted. Results are expressed as percentage of the time 0 (non-treated cells) colony number.

### 4.12. Preparation of Cell Lysates for Colorimetric Assay

For antioxidant measurements, yeast transformants grown up to OD_600_ 1.0 were harvested by centrifugation (4000× *g*; 10 min; 4 °C) (Allegra^®^ 6 Series Centrifuges, Beckman Coulter^®^), washed once with K+-citrate 50 mM pH 5.1 buffer, and resuspended to OD_600_ 1.0 in fresh growth media and incubated with 1mM H_2_O_2_ at 30 °C with orbital shaking for 60 min. Cells were then harvested by centrifugation (4000× *g*; 10 min; 4 °C) (Allegra^®^ 6 Series Centrifuges, Beckman Coulter^®^) and dry pellets were stored at −80 °C until analysis. Dry pellet was dissolved in phosphate buffered saline (PBS) and disrupted mechanically by vigorous agitation with acid washed glass beads for seven 1 minute intervals with cooling intervals between each agitation cycle. After disruption, cell lysates were cleared by centrifugation (7200× *g*; 10 min; RT) (VWR™ Micro 1207 Centrifuge) and the supernatants were used for assays. Prior to performing the assays, protein concentration of cell lysates was determined according to Bradford, using bovine serum albumin as a standard [41].

### 4.13. Preparation of Cell Lysates for Colorimetric Assay

The catalase activity was measured by the modified method of Goth [42]. This method is based on the measurement of H_2_O_2_ degradation in cell lysate, which occurs mostly by catalase activity, as it has one of the highest turnover numbers among all the enzymes. For catalase activity assay, 40 μL of supernatant was mixed with H_2_O_2_ (final concentration 65 mM) for the start of the reaction. Different dilutions of hydrogen peroxide (0–75 mM) were used for standards. The reaction was stopped after 5 min by addition of ammonium molybdate (final concentration 200 mM), and color development was measured spectrophotometrically in a plate reader at 405 nm (Anthos Zenyth 3100, Beckman Coulter^®^). One unit of catalase activity is defined as the amount of enzyme needed for degradation of 1 µmol of H_2_O_2/_min at 25 °C. Catalase activity was expressed as units of catalase per milligram of proteins in cell lysate (U mg^−1^).

### 4.14. Statistical Analysis

All the experiments were performed in biological and technical triplicates. Results were expressed as mean ± SEM of at least three independent experiments. Statistical analysis between groups was performed by two-way ANOVA and non-parametric Mann–Whitney test using the Graph Prism software (GraphPad Software, La Jolla, CA, USA). *p*-values < 0.05 were considered statistically significant.

## 5. Conclusions

This work unequivocally demonstrates that human AQP5 is involved in redox biology, facilitating the passive diffusion of H_2_O_2_ through cell membranes and contributing to cell proliferation and migration. Selective targeting of AQP5 may open new perspectives for anti-cancer drug development.

## Figures and Tables

**Table 1:** Nucleotide sequences of the PCR primers used for generating the mutants analyzed in the present study. Nucleotide sequences in turkish are for homologous recombination with pUG35; the bold sequence is a yeast Kozak sequence, while AQP5 sequences are shown in black. The codon changed in each primer is underlined. Non-tagged AQP5 mutations were made by transforming YSH1770 with *Bam*HI, *Sal*I, and *Hin*dIII digested pUG35 and two PCR products: one generated by AQP5UG35fw + a mutant rv primer the other by AQP5UG35rv + the corresponding mutant fw primer. GFP tagged versions were made in the same way except for using the AQP5UG35GFPrv primer instead of AQP5UG35rv. Non-tagged and GFP tagged wild type AQP5 were generated by transforming YSH1770 with *Bam*HI, *Sal*I, and *Hin*dIII digested pUG35 and PCR products generated by AQP5UG35 fw + AQP5UG35rv and AQP5UG35fw + AQP5UG35GFPrv, respectively.

AQP5UG35fw: 5’ ATACATAGATACAATTCTATTACCCCCATCCATAC**TAAGATAATT**ATGAAGAAGGAGGTGTGCTC 3’
AQP5UG35rv: 5’ ACAACACCAGTGAATAATTCTTCACCTTTAGACATTCAGCGGGTGGTCAGCTC 3’
AQP5UG35GFPrv: 5’ ACAACACCAGTGAATAATTCTTCACCTTTAGACATGCGGGTGGTCAGCTCCA 3’
H173AAqp5fw: 5’ ACCCTGGGCGCACTTGTCGGAATC 3’
H173AAqp5rv: 5’ GATTCCGACAAGTGCGCCCAGGGT 3’
H173WAqp5fw: 5’ ACCCTGGGCTGGCTTGTCGGAATC 3’
H173WAqp5rv: 5’ GATTCCGACAAGCCAGCCCAGGGT 3’
S156AAqp5fw: 5’ CGCCGCACCGCACCTGTGGGCT 3’
S156AAqp5rv: 5’ AGCCCACAGGTGCGGTGCGGCG 3’
S156EAqp5fw: 5’ CGCCGCACCGAACCTGTGGGCT 3’
S156EAqp5rv: 5’ AGCCCACAGGTTCGGTGCGGCG 3’
S183AAqp5fw: 5’ CACTGGCTGCGCAATGAACCCAGC 3’
S183AAqp5rv: 5’ GCTGGGTTCATTGCGCAGCCAGTG 3’
S183EAqp5fw: 5’ CACTGGCTGCGAAATGAACCCAGC 3’
S183EAqp5rv: 5’ GCTGGGTTCATTTCGCAGCCAGTG 3’

## Supplementary Materials

The following are available online at https://www.mdpi.com/2072-6694/11/7/932/s1, Figure S1: AQP5 expression and localization in transformed *S. cerevisiae* cells.
