# Supplementary material for: Human Aquaporin-5 Facilitates Hydrogen Peroxide Permeation Affecting Adaption to Oxidative Stress and Cancer Cell Migration
Source: Cancers (Basel). 2019 Jul 3;11(7):932. doi: 10.3390/cancers11070932 (PMC6678198; doi:10.3390/cancers11070932)
Supplement: Supplementary file 1 [file cancers-11-00932-s001.pdf]

Human aquaporin-5 facilitates hydrogen peroxide permeation affecting adaption to oxidative stress and cancer cell migration

Claudia Rodrigues [1,2], Catarina Pimpão [1,2], Andreia F. Mósca [1,2], Ana S. Coxixo [1,2], Duarte Lopes [1,2], Inês Vieira da Silva [1,2], Per Amstrup Pedersen [3], Fernando Antunes [4] and Graça Soveral [1,2,*]

Supplementary Materials

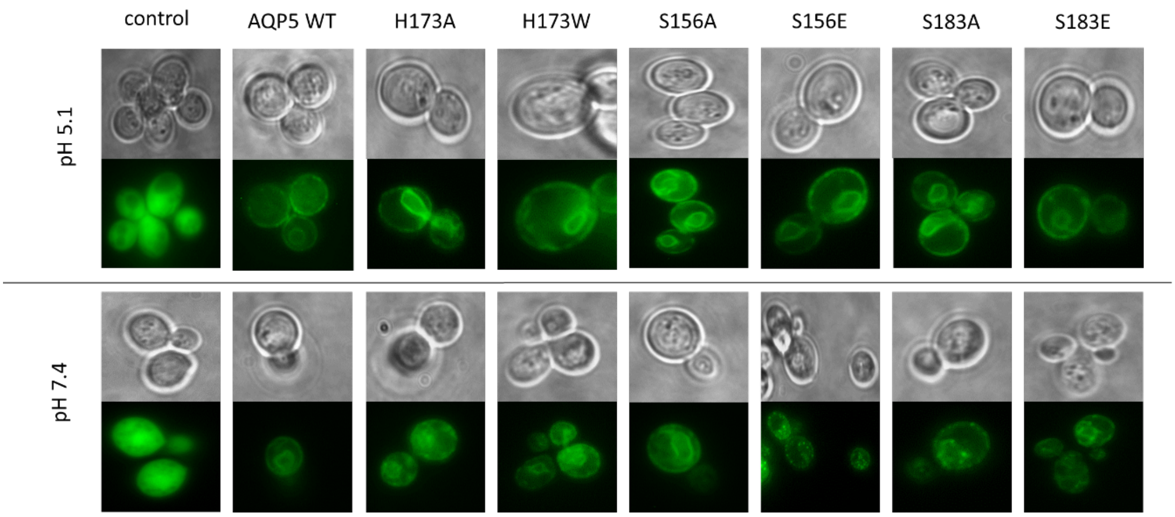

Figure S1. AQP5 expression and localization in transformed *S. cerevisiae* cells. Phase contrast (up) and epifluorescence (down) microscopy images (100x objective) of yeast aqy-null cells transformed with empty plasmid pUG35 (control cells with cytosolic GFP localization) and transformed with AQP5 WT and mutants at pH 5.1 (upper panel) and pH 7.4 (lower panel).
